# Vestibular Derangement and Motion Intolerance in VATER Association

**DOI:** 10.1155/2017/4507323

**Published:** 2017-05-22

**Authors:** Orit Samuel, Avi Shupak, Ayelet Eran, Dror Tal

**Affiliations:** ^1^Motion Sickness and Human Performance Laboratory, The Israel Naval Medical Institute, Israel Defense Forces Medical Corps, Haifa, Israel; ^2^Department of Otolaryngology-Head and Neck Surgery, Carmel Medical Center, Haifa, Israel; ^3^Unit of Otoneurology, Lin Medical Center, Haifa, Israel; ^4^The Bruce Rappaport Faculty of Medicine, The Technion, Haifa, Israel; ^5^Department of Radiology, Rambam Health Care Campus, Haifa, Israel

## Abstract

VATER association is a nonrandom occurrence of congenital malformations: vertebral defects, anal atresia, tracheoesophageal fistula, renal defects, and radial bone anomalies. We report the case of a 19-year-old man with a childhood diagnosis of VATER association, who presented to the motion sickness clinic with severe seasickness. We discuss the clinical and laboratory diagnosis of vestibular pathophysiology, which was confirmed by MRI of lateral semicircular canal and vestibule dysplasia. We suggest the possibility of vestibular involvement as part of the developmental field defect associated with VATER syndrome, which hitherto has rarely been reported.

## 1. Introduction

VATER association is a nonrandom occurrence of congenital malformations: vertebral defects, anal atresia, tracheoesophageal fistula, renal defects, and radial bone anomalies [1]. Its prevalence is 1 to 9 in 100,000 live births, more commonly in males. The phenomenon is a “developmental field defect” occurring in the blastogenesis phase and not just a statistical clustering. Temporal bone anomalies have been noted previously in the postmortem study of a VATER patient [2]. However, no pathophysiologic or imaging correlates were determined, and vestibular symptoms were therefore not designated as part of the syndrome.

Motion sickness is a physiological reaction to unfamiliar patterns of movement. A common form is seasickness. Nevertheless, some cases of intractable seasickness may be a consequence of vestibular pathophysiology.

We report a patient with a childhood history of VATER association, who presented with recalcitrant seasickness due to congenital vestibular malformation.

## 2. Case Report

A 19-year-old navy crewmember presented to the motion sickness clinic, complaining of severe seasickness. The patient had been sailing regularly for 4 months, with no improvement in his symptoms over time. He suffered from nausea, vomiting, loss of appetite, and depression in any sea condition. Bus, train, or rollercoaster rides produced similar symptoms. He reported no symptoms of dizziness, vertigo, or disequilibrium in his daily life.

The patient's past medical history included tracheoesophageal fistula, esophageal atresia, and imperforated anus. Spinal X-ray revealed 3 to 4 sacral vertebrae, with no coccyx. Based on these findings, the patient was diagnosed as suffering from VATER association. During early infancy, the patient underwent a number of corrective surgical procedures and was later asymptomatic.

Otoneurological examination revealed 1st-degree right beating spontaneous nystagmus, biphasic posthead shaking nystagmus with the first phase beating to the right, and bilateral corrective saccades on the head impulse test (L > R). Romberg's test, tandem walking, past pointing, and the Fukuda test were normal. No nystagmus was evoked by positional maneuvers or the Dix-Hallpike test. Audiometry demonstrated normal hearing.

Laboratory examination included videonystagmography. Gaze to the right produced a right beating nystagmus. Positional testing revealed intermittent direction changing apogeotropic nystagmus in three of the six testing positions, which was not of clinical significance. The Dix-Hallpike maneuver was within normal limits. The posthead shaking test produced a right and later left beating nystagmus. The caloric test revealed normal directional preponderance of 11% to the right and abnormal caloric weakness of 59% to the left. Otolithic function evaluated by the cervical vestibular evoked myogenic potentials test was within normal limits.

**Figure 1:**
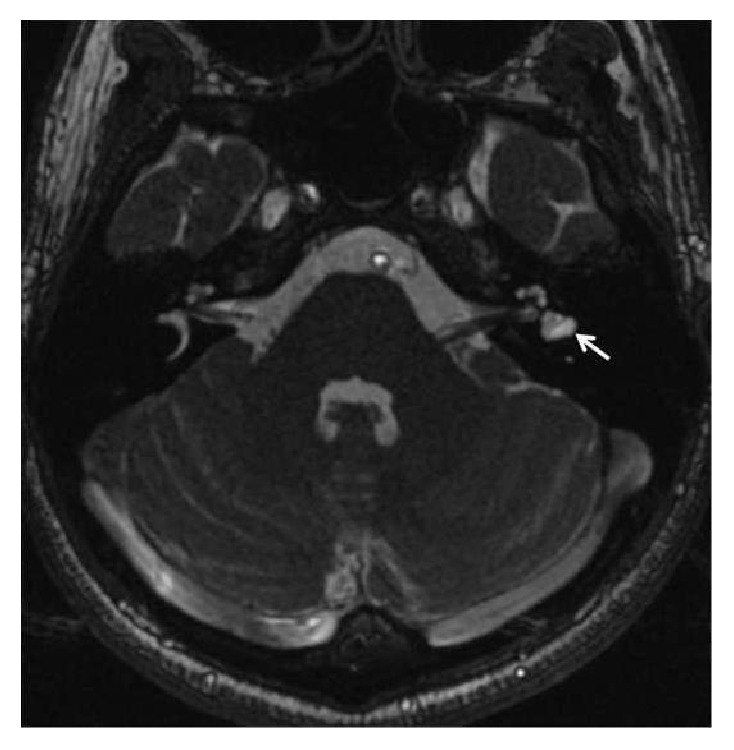
MRI of the patient showing a malformation of the left horizontal semicircular canal and vestibule forming a vestibular common cavity, compatible with the concept of lateral semicircular canal vestibule dysplasia (LCVD).

The patient was referred to high resolution temporal bone MRI study (Figure 1). This revealed a vestibular common cavity malformation of the dysplastic left horizontal semicircular canal and vestibule, compatible with* lateral semicircular canal vestibule dysplasia* (LCVD). These findings had previously been undiagnosed.

## 3. Discussion

The incidence of cochlear and vestibular abnormalities of the temporal bone was found to be higher in patients with congenital syndromes, compared with those who were nonsyndromic (20% versus 3%). These findings were not always associated with hearing loss [3, 4].

Autopsy of a 1-day-old newborn with VATER syndrome revealed various temporal bone pathologies [2]. Although the superior and posterior semicircular canals were intact, an anomalous course of the lateral canal was observed, and it also appeared to be an extended vestibular compartment rather than a curved canal. Furthermore, an abnormally high location of the utricle and saccule was demonstrated.

In our patient, the vestibular evaluation suggested bilateral peripheral vestibulopathy being more extensive on the left. While the bed-side head impulse test indicated malfunctioned vestibuloocular reflex responses to high-frequency stimuli, the videonystagmography caloric test demonstrated reduced performance of the left horizontal semicircular canal also in the low-frequency range. Although the MRI temporal bone study did not demonstrate anatomical malformation of the right inner ear or the 8th cranial nerve, bilateral functional involvement related to VATER association cannot be ruled out.

The lateral semicircular canal and vestibule dysplasia found on MRI matched the autopsy and CT findings of previous studies [2, 4].

The organs associated with the VATER syndrome develop during the first 4 to 8 weeks of gestation [5]. This is also the stage at which the vestibular system is being formed, and it is therefore possible that the processes which produce the VATER pathologies also result in vestibular anomalies.

In conclusion, this case demonstrates for the first time an association between VATER syndrome and vestibular pathologies using clinical and laboratory tests. We suggest that vestibular malformation and dysfunction might be underdiagnosed, due to early mortality in severe cases, or in the less severe due to masking of vestibular hypofunction by long-term central compensation processes. The severe seasickness experienced by our patient was probably the result of decompensation when exposed to challenging sea conditions.
